# Cross-Platform Array Screening Identifies *COL1A2*, *THBS1*, *TNFRSF10D* and *UCHL1* as Genes Frequently Silenced by Methylation in Melanoma

**DOI:** 10.1371/journal.pone.0026121

**Published:** 2011-10-20

**Authors:** Vanessa F. Bonazzi, Derek J. Nancarrow, Mitchell S. Stark, Ralf J. Moser, Glen M. Boyle, Lauren G. Aoude, Christopher Schmidt, Nicholas K. Hayward

**Affiliations:** 1 Oncogenomics Laboratory, Queensland Institute of Medical Research, Herston, Brisbane, Queensland, Australia; 2 SEQUENOM, Asia Pacific Office, Queensland Institute of Medical Research, Herston, Brisbane, Queensland, Australia; 3 Drug Discovery Group, Queensland Institute of Medical Research, Herston, Brisbane, Queensland, Australia; 4 Cancer Immunotherapy Group, Queensland Institute of Medical Research, Herston, Brisbane, Queensland, Australia; University of Texas MD Anderson Cancer Center, United States of America

## Abstract

Epigenetic regulation of tumor suppressor genes (TSGs) has been shown to play a central role in melanomagenesis. By integrating gene expression and methylation array analysis we identified novel candidate genes frequently methylated in melanoma. We validated the methylation status of the most promising genes using highly sensitive Sequenom Epityper assays in a large panel of melanoma cell lines and resected melanomas, and compared the findings with those from cultured melanocytes. We found transcript levels of *UCHL1*, *COL1A2*, *THBS1* and *TNFRSF10D* were inversely correlated with promoter methylation. For THBS1 and UCHL1 the effect of this methylation on expression was confirmed at the protein level. Identification of these candidate TSGs and future research designed to understand how their silencing is related to melanoma development will increase our understanding of the etiology of this cancer and may provide tools for its early diagnosis.

## Introduction

Aberrant epigenetic modifications are a feature of several human diseases, including cancer. While several forms of epigenetic modification are known to exist, so far DNA methylation is the only one shown to directly target DNA and to be frequently aberrant in many tumor types [1], [2]. By far the most common form of DNA methylation occurs via covalent modification of cytosine bases which precede a guanine residue (CpG). The reaction is catalysed by DNA methyltransferases [3] and results in a methyl group being added to the 5th position of the pyrimidine ring of cytosine. In mammals the majority of CpG sequences are methylated, with the exception of CpG-rich stretches present within the 5′ regulatory components of many genes, termed CpG islands [4]. There is strong evidence suggesting an inverse relationship between the presence of CpG island methylation and the level of target gene expression [5], although this suppression is not always evident. Both hypermethylation of CpG islands located in the promoters of tumor suppressor genes (TSGs) and global hypomethylation seem to play an important role during cancer development. Often TSGs are not primarily inactivated through mutation or deletion, but rather through targeted CpG island methylation.

Melanoma genomics studies have identified a large number of chromosomal loci that show repeated loss of heterozygosity (LOH), highlighting widespread chromosomal instability [6], [7]. Additionally, aberrant promoter methylation may also occur and lead to inactivation of TSGs which play a role in progression to malignancy. During melanomagenesis, well-known TSGs, such as *PTEN*, *CDKN2A/p16INK4A* and *RASSF1A* often have expression reduced through CpG island methylation [8], [9], [10]. In the last decade, several studies have assessed genome-wide methylation using the DNA methyltransferase inhibitor 5-aza-2-deoxycytidine (5AzadC) and identified *TSPY*, *HOXB13* and *SYK* as novel TSGs in melanoma [11], [12], [13].

In a previous study, we combined 5AzadC treatment with Trichostatin A (TSA), an inhibitor of class I and II histone deacetylase enzymes, and conducted a microarray-based analysis on a panel of melanoma cell lines identifying eight highly ‘reactivated’ genes (expression fold change >4), not previously known to be epigenetically silenced in melanoma [14]. For five of these genes there was no prior evidence of inactivation by promoter methylation in any other cancer type. Follow up of these genes was carried out in a larger panel of melanoma cell lines, in addition to fresh tumors and melanocyte cultures, using the highly sensitive Sequenom Epityper assay [15], [16] and correlated with microarray based gene expression levels. Four genes: *PPP1R3C* (protein phosphatase 1, regulatory (inhibitor) subunit 3C), *ENC1* (ectodermal-neural cortex 1), *RARRES1* (retinoic acid receptor responder also known as tazarotene induced gene 1, TIG1) and *TP53INP1* (tumor protein p53 inducible nuclear protein 1), had mRNA levels that were inversely correlated with promoter methylation (>40–60% of CpG sites) in 35–59% of melanoma cell lines and 6–25% of the fresh tumors.

In order to identify additional epigenetically silenced genes implicated in melanocytic neoplasia, we have generated new data from 11 melanoma cell lines using Illumina Infinium Methylation27 arrays [17]. In order to provide a more complete picture of methylation in melanoma, these data were integrated with our previous constitutive mRNA expression [18], [19] and post-demethylation treatment expression data [14] to identify a list of potential genes for assessment by comprehensive promoter methylation analysis using the Epityper system in an extended panel of melanoma cell lines and tumors.

## Results

### Description of a new pipeline to identify novel candidate gene CpG islands methylated in melanoma

In our previous study, we used a microarray-based strategy in a panel of 12 melanoma cell lines treated with 5AzadC and TSA as an initial screening approach. Select candidate genes were followed up using the Epityper assay in a much larger panel of melanoma cell lines, as well as a panel of fresh-frozen melanoma samples, normal melanocyte cultures, and cell lines from other cancer types. We identified four genes, *PPP1R3C*, *ENC1*, *RARRES1* and *TP53INP1* that were not previously known to be silenced by DNA methylation in melanoma [14].

In order to use more robust criteria to select the genes epigenetically silenced during the development of melanocytic tumors, we have generated new data on 11 melanoma cell lines using Illumina Infinium Methylation arrays. The Infinium Methylation chips interrogate 27,578 CpG loci covering more than 14,000 genes. We screened 11 melanoma cell lines from our pilot study [14], which we compared to pools of melanocytes from several donors.

**Figure 1:**
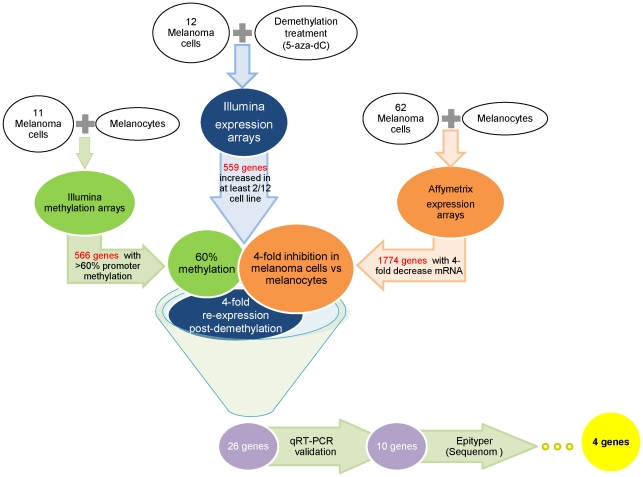
Flow through of the cross-platform array integration.

These new data were then integrated with previous data-sets of global mRNA expression [18], [19] and expression post-demethylation treatment [14] in order to focus on identifying additional candidate TSGs down-regulated through promoter methylation. Genes were further filtered to identify those in which ≥60% methylation correlated with a 4-fold decrease in mRNA levels in at least 2 samples, together with an average post-demethylation re-expression fold-change of >4 across the panel of 11 melanoma lines (**Figure 1**). This gave a set of 26 genes, from which we then removed oncogenes (*ADM*, *ENPP2*, *RAC2*, *SERPINE1*), genes we had previously identified (*PPP1R3C*), those without a described function e.g. annotated as “orf” (*C10orf116*, *SLC25A38*, *CCDC109B*), and false positives on the Infinium Methylation chips (i.e. multiple instances where cell lines were methylated on the HumanMethylation27 BeadChip but showed no re-expression with 5AzadC+TSA treatment; *EEF1A2*, *HSPA2*).

These additional filters resulted in 12 genes remaining for initial follow up (*COL1A2*, *CRABP2*, *CRIP1*, *FAM46B*, *GATA2*, *IGFBP4*, *LOX*, *RGC32*, *THBS1*, *TNFRSF10D*, *UCHL1* and *VAMP8*). The genes were further filtered by validating the differences in their expression levels pre- and post-treatment. Transcript levels of these 12 genes were assessed by qRT-PCR in the 5 cell lines with the highest expression differences before and after treatment. Selecting only the genes with a >4–fold average qRT-PCR expression change and the presence of CpG islands in the promoter gave a final list of 10 genes: *COL1A2*, *CRABP2*, *CRIP1*, *GATA2*, *IGFBP4*, *LOX*, *RGC32*, *THBS1*, *TNFRSF10D* and *UCHL1*. While the absolute value of the fold-changes varied somewhat between the two methodologies, there was generally good agreement between the microarray and qRT-PCR results (**Figure S1**). Importantly, for each of these 10 genes both techniques showed a >4–fold average change in expression after drug treatment.

We then quantitated the degree of methylation of these 10 candidate genes using mass spectrometry of base-specific cleaved amplification products (Sequenom Epityper assay [15], [20]). This technique was applied to a panel of 45 melanoma cell lines, 30 fresh melanoma tumor samples, and 2 independent pools of cultured melanocytes for comparison as the non-malignant control cell type. Each gene promoter was divided into one or more amplicons, within a region comprising 2500 bp upstream of the transcription start site and covering the CpG islands described in the UCSC genome browser. These amplicons were then amplified by PCR (T**able S1**) and subjected to the Epityper assay. Three genes were not considered further as they either failed to give any analysable data, probably due to the high CpG density of the region (*CRABP2*), or showed no differences in methylation profiles between melanocytes and the melanoma cell lines (*LOX* and *RGC32*).

For each gene, the amplicons included in the analysis were those with different average methylation values of >20% in the entire panel of melanoma cell lines and with <10% methylation in melanocytes (**Figure S2**). These informative CpG sites were then scored in each amplicon defining the CpG island for each gene. Only the CpG sites presenting high methylation ratios (as defined below) in melanoma cell lines were averaged for the final percentage of methylation (% of methylation). This allowed grouping of the melanoma cell lines following their % of methylation: no/low methylation (0–20%), medium (20–50%) and high methylation (>50%). We then defined the average % of methylation for each gene as the average value across the melanoma cell line panel which was then compared to melanocytes.

Integrating the degree of methylation and the level of mRNA expression, as assessed by previous Affymetrix microarray analysis [19] gave high correlation coefficients (Spearman coefficients = −0.75/−0.82/−0.6/−0.52 respectively, p<0.005) for four genes: *COL1A2*, *THBS1*, *TNFRSF10D* and *UCHL1*.

### Expression of the COL1A2, THBS1, TNFRSF10D and UCHL1 genes is inhibited by CpG island methylation in melanoma cell lines

Following 5AzadC+TSA treatment, *COL1A2* and *THBS1* transcript levels were increased by an average of 58-fold in 9 of 12 cell lines and 40-fold in 8 of 12 cell lines respectively (**Table S3**). For *TNFRSF10D*, 5 cell lines showed a 5-fold average increase in expression.

In melanoma cell lines, the average % of methylation for these genes was 24%, 31% and 66% respectively. The methylation levels of these genes in the melanocyte pools were close to background (between 2 to 9%).

The 5′UTR regions around the transcription start sites of *COL1A2* and *THBS1* were both divided into 5 amplicons, while 4 amplicons were designed to cover the 5′UTR and first exon of *TNFRSF10D* (F**igure S2**). For each of the three genes, the amplicons closest to the transcription start (within 1000 bp) showed high levels of methylation in the melanoma samples, which inversely correlated with mRNA expression (T**able S1**). **Table S4** summarizes the number of CpG sites scored for the definition of the % of methylation for each gene (19, 15, 37 and 19 for *COL1A2*, *THBS1*, *TNFRSF10D* and *UCHL1* respectively).

**Figure 2:**
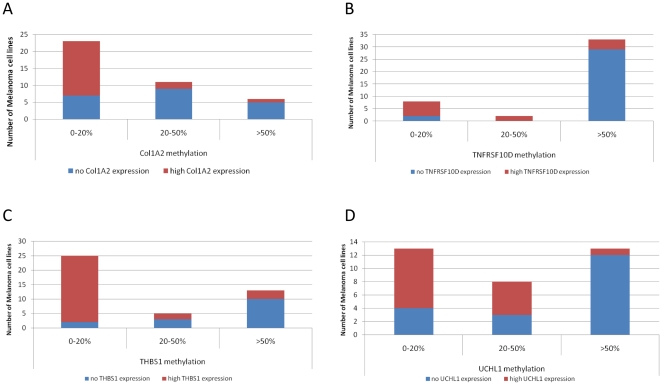
Distribution of the melanoma cell lines according to their methylation status for a. COL1A2, b. TNFRSF10D, c. THBS1, d. UCHL1. The melanoma cell lines were grouped following their mRNA expression level, no or high expression. Methylation levels were determined by Epityper assay.

Over fifty percent (21/40) of the melanoma cell lines had no *COL1A2* mRNA expression, which correlated with a high degree of *COL1A2* promoter methylation in 67% of this subset. The other 19 cell lines with detectable *COL1A2* mRNA expression all showed <20% of methylation, with the exception of 3 lines (MM415, MM229 and D05). (**Figure 2a** **- Figure S3a**).

For *TNFRSF10D*, 31 of 43 (72%) melanoma cell lines had no mRNA expression. Of the 31 lines not expressing *TNFRSF10D*, 28 (90%) showed >60% of methylation (**Figure 2b** **- Figure S3b**). For *THBS1*, 15 of 43 (35%) melanoma cell lines had no mRNA expression. Eight of the 15 (53%) lines with no *THBS1* mRNA showed a high >50% methylation (**Figure 2c** **- Figure S3c**).

Following 5AzadC+TSA treatment, *UCHL1* expression was increased by an average of 18-fold in 10 out of 12 cell lines. For the Epityper assay, the region around the 5′UTR and transcription start site of *UCHL1* was divided in to 4 amplicons, also covering both exons 1 and 2 (F**igure S2**). While amplicons 2 and 6 did not show any differential methylation between melanoma cell lines and melanocytes (T**able S1**), amplicons 4 and 8 showed specific profiles of methylation in melanoma cell lines inversely correlated with mRNA expression (Spearman = −0.53). A majority (56%) of the melanoma cell lines had no *UCHL1* mRNA expression, which correlated with a high degree of *UCHL1* promoter methylation in 79% of them (5% of methylation in melanocytes compared to an average of 42% in melanoma cell lines, range = 29–95%). The melanoma cell lines with high *UCHL1* mRNA expression have <20% methylation (**Figure 2d** **- Figure S3d**).

**Figure 3:**
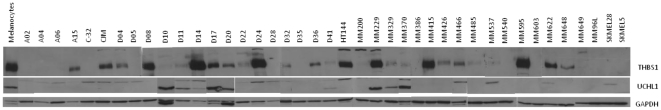
Western blot analysis of THBS1 and UCHL1 protein expression.

Next, we assessed protein levels for THBS1 and UCHL1 (since robust target-specific commercial antibodies are available) by western blot analysis (**Figure 3**). There was a high correlation between mRNA and protein expression (Pearson = 0.84 and 0.51 for UCHL1 and THBS1 respectively), and an inverse correlation between protein expression and the methylation profile (Spearman = −0.52 and −0.33 for UCHL1 and THBS1 respectively).

### Confirmation of candidate gene promoter methylation in fresh-frozen melanoma tumors

Simultaneously, in the Epityper assay, we included 30 fresh-frozen melanoma tumor samples and found that 13%, 15% and 30% of them respectively were methylated for *COL1A2*, *THBS1* and *TNFRSF10D* (**Table 1**). On average, the *COL1A2* and *TNFRSF10D* promoters appeared >10–fold more methylated in melanoma cell lines compared to melanocytes, and 6-fold higher in the fresh tumors (**Figure S4**). *THBS1* promoter methylation levels were 3.5 and 2 times higher in melanoma cell lines and tumors respectively.

**Table 1:** Summary of *COL1A2*, *THBS1*, *TNFRSF10D and UCHL1* methylation in melanocytes, melanoma cell lines, fresh-frozen melanoma tumors and other cancer cell lines.

% of methylation in melanoma	other cancer cell lines							
	In melanocytes	In MM cell lines	MM/mel	In Fresh tumors	FT/Mel	esophageal cancer	glioma	colon cancer
Col1A2	2%	24%	**10.48**	13%	**5.76**	65%	5%	**92%**
THBS1	9%	31%	**3.43**	15%	**1.79**	2%	4%	43%
TNFRSF10D	5%	66%	**13.03**	30%	**6.05**	2%	33%	2%
UCHL1	5%	42%	**8.10**	21%	**4.05**	**82%**	5%	**90%**

Ratios to melanocytes are presented for the % of methylation in melanoma cell lines and melanocytes (MM/mel) and the fresh-frozen tumors (FT/mel).

MM malignant melanoma.

mel melanocytes.

FT fresh-frozen tumors.

The *UCHL1* promoter appeared to be 8-fold more methylated in melanoma cell lines compared to melanocytes, and 4-fold more methylated in the 30 fresh tumors compared to the same control. Since mRNA levels were not assessed in the tumors, we are unable to correlate the proportion of methylation with expression in these samples. The observed methylation rates in tumors are lower than in the melanoma cell lines, as expected due to stromal contamination, which will likely have the effect of decreasing the observed overall percentage of methylation of the DNA assessed.

### Methylation of candidate gene promoters in other cancer types

In order to assess the possible specificity of methylation of the four candidate genes to melanoma, we repeated the same Epityper assays on a limited number of cell lines from cancers of the colon, esophagus and brain (glioma). These different cancer types were assessed to determine whether the candidate genes might play a more general role in tumor suppression. Two cell lines from each tumor type were assessed for their methylation status for *COL1A2*, *THBS1*, *TNFRSF10D* and *UCHL1*. Using the same cut off as for the melanoma cell lines and tumors, the percentage methylation for the *COL1A2* and *UCHL1* CpGs islands was 65% and 82% in the esophageal cancer cell lines respectively and 92% and 90% in the colon cancer cell lines respectively. While *THBS1* was only methylated in the colon cancer cell lines (43% methylation), *TNFRSF10D* appeared to be the only gene methylated in the glioma lines (33%) (**Table 1**).

## Discussion

The objective of this study was to combine different array platforms to strengthen the identification of novel TSGs inactivated by promoter methylation in melanoma. Selection of candidate genes was based on reduced expression in a panel of melanoma cell lines which correlated with a high methylation profile, and lack of the same observation in melanocyte cultures. Using these criteria we identified and subsequently confirmed four genes silenced by DNA methylation in melanoma. We found 24%, 31%, 66% and 42% of cell lines and 13%, 15%, 30% and 21% of tumors were methylated for *COL1A2*, *THBS1*, *TNFRSF10D* and *UCHL1* respectively.

Each of these four genes had previously been linked to melanoma. Muthusamy *et al.* and Koga *et al.* [12], [21] identified *COL1A2* as methylated in 35% (7/20) to 89% (16/20) of melanoma tumor samples respectively. In their systematic methylation profiling of several human cancer cell lines, Paz *et al.* reported reactivation of *THBS1* expression following 5AzadC treatment in all 18 melanoma cell lines analysed [22]. Liu *et al.* [23] showed *TNFRSF10D* promoter methylation in 85% of their melanoma cell lines (17/20) and 80% of their fresh melanoma tumor samples (32/40). In their study of the molecular effects of low dose of 5AzadC (Decitabine) on 8 melanoma cell lines, Halaban *et al.* [24] presented evidence for changes in expression of *COL1A2, TNFRSF10D, THBS1* and *UCHL1*. Here, we further document the link between these genes and melanoma by confirming the correlation between methylation and expression of these genes in a larger panel of melanoma cell lines. In our study, we assessed 45 melanoma cell lines, 30 fresh tumor samples and correlated methylation status and mRNA expression levels to observations made in pooled melanocytes. Furthermore, while the majority of the previous methylation studies were based on the analysis of gene re-expression post-5AzadC treatment but not on assesment of promoter methylation *per se*, we present here a study design which goes beyond 5AzadC treatment to include a precise CpG methylation profile via the Epityper assay, a sensitive and high-throughput method for DNA methylation analysis.

Within the large collagen family, collagen type I is the most abundant, structural component of healthy connective tissue and consists of a heterotrimer of two α1 (COL1A1) and one α2 (COL1A2) chains. Cellular p53 negatively regulates COL1A2 through TGF-β signalling in normal dermal fibroblasts [25]. Evidence for *COL1A2* aberrant promoter methylation has been described in different cancer cells such as breast cancer, medulloblastoma, hepatoma, colorectal cancer [26], [27], [28] and more recently in melanoma [12], [21]. Sengupta *et al.* describe the possible advantages a decrease in collagen synthesis may confer on cancer cells, including faster cell growth and increased tumorigenic potential [28].

*THBS1* (also known as *TSP-1*) encodes the glycoprotein thrombospondin, which is generally considered a tumor suppressor and mediates cell-to-cell and cell-to-matrix interactions important for platelet aggregation and angiogenesis [29].

Down-regulation of *THBS1* by methylation has been described in several cancer types such as neuroblastoma [30], [31], colorectal [32], [33], [34] and stomach cancers [35], [36], [37]. Promoter hypermethylation of THBS1 was detected in brain metastases of solid tumors such as, melanoma, lung, ovarian and breast carcinomas [38] and more recently associated with bad prognosis in penile squamous cell carcinoma [39].

The protein encoded by *TNFRSF10D* (TRAIL4, DcR2) is a member of the TNF-receptor superfamily containing an extracellular TRAIL-binding domain and a truncated cytoplasmic death domain. This receptor does not induce apoptosis but has been shown to play an inhibitory role in TRAIL-induced apoptosis [40]. Like all the other genes encoding TRAIL receptors, *TNFRSF10D* expression is directly regulated by p53 and regulates cellular chemosensitivity [41]. *TNFRSF10D* promoter hypermethylation has been described as a mechanism of inactivating this gene in several cancer types [42], [43], [44].

The current study details *UCHL1* (ubiquitin COOH-terminal esterase L1) inactivation by promoter methylation in melanoma. This gene encodes a peptidase activator of the ubiquitin-dependent protein degradation pathway. Originally identified in neurons and in cells of the diffuse neuroendocrine system, mutations in this gene have been associated with Parkinson disease [45], [46]. UCHL1 has been attributed important roles in multiple cellular processes such as proliferation, cell cycle, apoptosis and intra-cellular signalling. Its role in tumorigenesis, TSG or oncogene, seems to be dependent on the tumor type [47], [48]. *UCHL1* methylation has been reported in multiple tumors [49], such as esophageal [50], gastric [36], [51], renal [52], prostate [53], head and neck squamous [47], ovarian [54], hepatocellular and colorectal cancers [49], [55]. Some studies even suggested the use of *UCHL1* methylation as a biomarker for diagnosis and prognosis of certain tumors [50], [55], [56]. Li *et al.* recently showed in nasopharyngeal carcinoma cell lines that UCHL1 was a member of the p53/p14ARF/MDM2 complex [48]. Through its deubiquitinating activity, UCHL1 is involved in the stabilisation of p53 and p14ARF. In parallel, it also decreases the amount of MDM2 by promoting its degradation through ubiquitination. A reduction in UCHL1 expression has previously been associated with poor survival in melanoma [57]. Our study confirms that this is likely to occur via hypermethylation of the *UCHL1* regulatory region.

Interestingly, all four genes (*COL1A2, THBS1, TNFRSF10D* and *UCHL1*) we identify here as methylated in melanoma, encode components that fit within the p53 ontology pathway. Moreover, two of the genes we had identified in a previous methylation study are also associated with p53 function, either being induced by p53 (*ENC1*) or interacting with p53 (TP53INP1) [14]. In melanoma, direct inhibition of p53 by mutation is relatively infrequent (see [58] for review). Our finding of six candidate TSGs linked to p53 function that are subject to methylation in melanoma might indicate alternative mechanisms by which these cells abrogate p53 downstream signalling in this tumor type. Further functional validation of these methylated genes is necessary to confirm their importance in melanocytic neoplasia, their candidacy as potential TSGs, as well as their possible relationship to p53 status.

In summary, we have used a multiplatform integrative approach to identify a short list of robust methylated genes in melanoma. We confirm previous reports that *COL1A2*, *THBS1*, *TNFRSF10D* and *UCHL1* are highly methylated in melanoma, thus providing further evidence that these genes are highly important in melanocytic neoplasia.

Others have used somewhat similar integrative approaches [59], [60]. Sjaputera et al. [60] compared methylation array data with expression data with an arbitrary methylation cut off; and Loss et al. [59] integrated methylation and expression data followed by logistic regression to identify the most significantly affected genes. However, they did not include re-expression data following 5azadC treatment, or quantification of the degree of methylation using the Epityper or a similar assay.

While these other two studies were done on different cancer types (breast cancer and lymphomas), one common methylated gene, *COL1A2* was identified. Taken together, this suggests that *COL1A2* is a gene for which methylation is more generally associated with tumorigenesis across different cancer types.

## Materials and Methods

### Cell Culture

A panel of 12 melanoma cell lines derived from primary cutaneous melanomas or their metastases were used [14]. All cell lines were cultured in RPMI 1640 supplemented with 10% fetal bovine serum as described previously [19], [61]. Primary human melanocytes were obtained from neonatal foreskins and cultured in 10% heat-inactivated fetal calf serum (CSL, Melbourne, Australia) in RPMI-1640 medium supplemented with 100 U/ml penicillin, 100 µg/ml streptomycin, 3 mM HEPES with the addition of 6 ng/ml cholera toxin and 16.2 nM phorbol 12-myristate 13-acetate (PMA) (Sigma Chemical Co., St. Louis, MO) as previously described [62]. All tissue was taken with written informed consent under a protocol approved by the Queensland Institute of Medical Research Human Research Ethics Committee (HREC), approval number H0311-084 (P726).

The 2 colorectal cancer cell lines Co115 and LIM 2405, esophageal cancer cell lines OE19 and OE33, glioma cell lines T46 and T50 were purchased from the American Type Culture Collection (http://www.atcc.org/).

### DNA extraction, Bisulfite Conversion and Illumina Infinium Methylation27 Array Hybridization

QIAGEN DNeasy Blood and Tissue Kits were used to isolate genomic DNA from cells in log phase growth as per the manufacturer's instructions (QIAGEN,Hilden). All samples were run on an Agilent Bioanalyzer (Agilent, CA, USA) using a DNA 12000 LabChip kit to check for DNA integrity, purity and concentration. 500 ng of genomic DNA from 11 melanoma cell lines and a reference pool of melanocytes derived from several donors were bisulfite treated using an EZ-96 DNA methylation kit (Zymo Research, CA, USA) as per the manufacturer's instructions. They were then hybridized to Infinium Methylation BeadChips (Illumina,CA,USA) containing 27,578 CpG loci covering more than 14,000 genes [17]. All reagents and procedures for washing, detection and scanning were performed according to the BeadStation 500× system protocols (Illumina, CA, USA).

### Beadarray methylation analysis

Percent methylation (beta) was calculated from the ratio of fluorescent signal intensities of the methylated (M) and unmethylated (U) alleles, for each sample at each specific CpG site, using the equation beta = Max(M,0)/[Max(M,0)+Max(U,0)+0]×100. On this scale unmethylated sites are represented by beta values close to zero, while heavily methylated sites show values approaching 100%. We then expressed each site specific melanoma cell line methylation value as a delta difference, compared to that of melanocytes. In this way negative values represented a cell line and site specific decrease in methylation, while positive scores indicated a relative increase in the degree of methylation. The provided manifest file linked individual methylation sites to official gene symbols, which we used to associate the methylation data to that of mRNA expression and demethylation, as described below. The data are MIAME compliant and have been deposited in Gene Expression Omnibus database (http://www.ncbi.nlm.nih.gov/geo/) under accession number GSE28356.

### Microarray gene expression profiling

These data (GSE7127) were generated as part of previously published studies [18], [19]. Briefly, 5 µg of total RNA from 35 melanoma cell lines and one melanocyte foreskin pool were applied to Eukaryotic One-cycle Target Labelling and Control Reagents kits, according to the manufacturer's instructions (Affymetrix, CA, USA), and 15 µg of the resulting fragmented cRNA mixtures was then hybridized to an Affymetrix Human Genome U133 Plus 2.0 Array for 16 hr at 45°C. Chips were then washed and streptavidin phycoerythrin post-stained, before scanning on an Affymetrix GeneChip Scanner 3000. Relative expression values for each probe set were generated from the raw image data files using the Affymetrix PLIER algorithm in ArrayAssist 1 Version 4.20 (Stratagene). The resulting data were imported into GeneSpring GX v7.3 (Agilent Technologies) where data values less than 0.1 were set to 0.1 prior to log transformed to base 2. The expression values were then centrally normalized to the median expression value for each sample and the median expression value for each probe set. For the current study, expression data for each of the 11 melanoma cell lines for which we have Illumina Infinium Methylation Array were expressed as a ratio, per gene symbol, compared to that of the included melanocyte cell line. In this way we are able to match a single methylation value to an expression estimate for each of the 11 cell lines with matched data.

### 5AzadC demethylation profiling

These data were generated as part of a previously published study [14] (GSE32492). Briefly, cells were split to 20% confluence 24 hr prior to commencing a 3-day treatment with either 5 µM of 5AzadC (Sigma) from 100 mM 50% acetic acid dissolved stock, or mock treated with the same volume of phosphate buffered saline (PBS)/50% acetic acid. The 3-day incubation was followed by a 4-hr incubation with 300 nM TSA (Sigma) prior to total RNA extraction from cells in log phase growth (RNeasy Midi-kits – Qiagen, Hilden) and on-column DNase digestion. Samples with an Agilent Bioanalyzer (Agilent, CA, USA) determined RNA integrity number (RIN) of >8.0 were used for microarray analysis. Biotinylated cRNA were prepared from 500 ng of total RNA using an Illumina TotalPrep RNA Amplification Kit (Ambion, TX, USA) and 1500 ng was hybridized to Sentrix Human-6 Expression version 2 BeadChips (Illumina, CA, USA) prior to washing, detection, and scanning according to the BeadStation 500GX system protocols (Illumina).

Expression profiles generated for each cell line before and after drug treatment (expressed as a fold-change ratio) showed that across the panel of 12 cell lines a total of 8,144 non-redundant genes were re-expressed with >2-fold change after treatment (between 1,457 and 3,386 genes in individual samples). Genes reactivated in all 12 cell lines were removed from further analysis since they are likely to represent cellular stress response to 5AzadC treatment, or promoter demethylation of genes normally silenced within the melanocytic lineage.This filtering left 3125 genes to be considered from cross-analysis with the Beadarray27 methylation and U133 Plus 2.0 mRNA expression data described above.

### Combining mRNA profiling, demethylation and Beadarray27 data across 11 melanoma cell lines

Fold-change expression profiling data (compared to melanocyte pool), fold-change demethylation data (compared to matching untreated cell line) and Illumina methylation profiling data (delta % methylation compared to the melanocyte pool) for each of the 11 cell lines for which all data were available, were imported into Microsoft Excel and linked via official gene symbol (HUGO Gene Nomenclature Committee). Data filters were then applied to each of the three data types in order to identify genes with clear evidence of methylation (through both the presence of increased methylation values, and with increased expression following demethylation) and evidence of reduced expression compared to the melanocyte pool. Genes symbols were filtered to identify those in which at least 2 samples showed ≥60% methylation (Beadarray27) correlated to an average post-demethylation re-expression fold-change of >4 and 4-fold mRNA global decrease across the panel of 11 melanoma lines. From this set of 26 genes, after removing oncogenes (ADM, ENPP2, RAC2, SERPINE1), genes we had previously identified (PPP1R3C), those without a described function e.g. annotated as “orf” (C10orf116, FLJ20551, FLJ20647), and the presence of false positives on the Infinium Methylation chips (i.e. multiple instances where cell lines were methylated on the Beadarray27 but showed no re-expression with 5AzadC treatment; EEF1A2, HSPA2), 16 genes remained for initial follow up (COL1A2, CRABP2, CRIP1, FAM46B, GATA2, IGFBP4, LOX, RGC32, THBS1, TNFRSF10D, UCHL1, ALDOC, COL12A1, GALM, VAMP5 and VAMP8). Of these, 12 genes (COL1A2, CRABP2, CRIP1, FAM46B, GATA2, IGFBP4, LOX, RGC32, THBS1, TNFRSF10D, UCHL1 and VAMP8) were subject to further validation as described below.

### Quantitative RT–PCR

mRNA extraction & expression array data were obtained as previously described [14], [18], [19]. To confirm the validity of the microarray expression data, the mRNA levels were assessed by quantitative reverse transcriptase–polymerase chain reaction (qRT–PCR) (see Table S2 for primer sequences) in the 5 cell lines with the highest expression differences before and after 5AzadC treatment. First-strand cDNA synthesis was performed with 3 µg total RNA for each sample in a total volume of 20 µl using Superscript III reverse transcriptase and random primers (Invitrogen, CA, USA). Subsequent PCR reactions were carried out on a Corbett RotorGene 6000 (Corbett Research, Australia) using SYBR Green RT–PCR Master Mix (Applied Biosystems, Foster City, CA). CLTA (clathrin light chain mRNA) was chosen as the normalization control transcript based on minimum variation across the cell lines as assessed by microarray [14].

### EPITYPER Assay

The Sequenom EpiTYPER assay is based on in-vitro transcription and base-specific cleavage of a PCR amplicon and the subsequent analysis of the resulting RNA fragments by MALDI-TOF mass spectrometry [15], [20].

EZ-96 DNA methylation kits (Zymo Research, CA, USA) were used for bisulfite treatment of 1 µg of genomic DNA from 44 melanoma cell lines, 1 nevus cell line, 2 colorectal cancer cell lines (Co115 and LIM 2405), 2 esophageal cancer cell lines (OE19 and OE33), 2 glioma cell lines (T46 and T50), and 30 fresh-frozen melanoma tumors. DNA from pools of melanocytes was used as reference. Each gene promoter was divided into several amplicons (Table S1). The target regions were then amplified using the primer pairs containing a T7-promoter tag (forward: 5′-AGGAAGAGAG-fw primer-3′, reverse: 5′-CAGTAATACGACTCACTATAGGGAGAAGGCT-rev primer-3′) to allow further in vitro transcription. One microliter of modified DNA was used for the PCR reactions carried out in a total volume of 5 µl. Unincorporated dNTPs were dephosphorylated by incubation at 37°C for 40 min in the presence of shrimp alkaline phosphatase (SAP) (Sequenom). Two microliters of this SAP-treated PCR mixture were used as a template in a 7 µl transcription reaction containing RNase A and T7 polymerase (Sequenom). Transcription and digestion were performed simultaneously at 37°C for 3 h. After the addition of 20 µl of H_2_O and 6 mg of CLEAN resin (Sequenom), 22 nl of the cleavage reactions were dispensed onto silicon chips preloaded with matrix (SpectroCHIPS, Sequenom). Mass spectra were collected using a MassARRAY mass spectrometer (Bruker-Sequenom) and analysed using proprietary peak picking and signal-to-noise calculations (Sequenom Epityper v1.0.5).

The relative amount of methylation (% methylation) was determined by comparing the signal intensities between the mass signals of methylated and non-methylated template.

For data analysis only unique CpG units (units can contain one or more consecutive CpG dinucleotides) are included. CpG units overlapping with other cleavage fragments in the mass spectrum were excluded from data analysis. CpG methylation ratios were filtered using an uncertainty threshold of 10%. Only data values (CpG methylation ratios) with an estimated error smaller than 10% were included in the analysis. This filtering ensured that only precise data values were used for downstream calculation.

For each gene, the amplicons presenting no significant difference in methylation between melanoma cell lines and melanocytes were dropped from the analysis (Figure S2). In each amplicon, the informative CpG sites were then scored defining the CpG island for each gene. Only the CpG sites presenting high methylation levels in melanoma cell lines were included in the final percentage of methylation. The average % of methylation for each gene was then defined as the average value across the melanoma cell line panel which was compared to melanocytes.

### Statistical Analysis

Spearman test was applied for the correlation between mRNA expression and methylation of the genomic region assessed. A t-test (t = r/Sr) was performed to obtain the significance (Sr = (1−r^2^)/n) of the Spearman coefficient.

### Western blot analysis

Total cell lysates from 1.10^7^ cells were generated as previously described [63]. Samples (30 µg protein) were resolved by 10% SDS-polyacrylamide gel electrophoresis, and transferred to nitrocellulose membranes. Antibodies raised against the following proteins were used for Western blotting: anti-THBS1 (ab88529, 1 in 1,000 dilution; Abcam, Cambridge, UK), anti-UCHL1, (1 in 1,000 dilution; Sigma Prestige antibodies, St. Louis, MO), and anti-GAPD (1 in 5,000 dilution; R&D Systems, Minneapolis, MN). Detection was performed using the appropriate peroxidase-conjugated secondary antibody and a Western Lightning chemiluminescent reagent plus kit (PerkinElmer LAS, Inc., Boston, MA).

## Supporting Information

Figure S1 **Comparative expression between microarray and qRT-PCR for 12 candidate genes.** Plotted are the mean fold-change values for 5 cell lines with the highest expression differences before and after 5Aza-dC treatment.
(PDF)

Figure S2 **UCSC browser for localisation of the amplicons used for the Epityper assays.** The alignments show the localisation of the Illumina probes.
(PDF)

Figure S3 **Distribution of the melanoma cell lines according to their methylation status for a. COL1A2 mRNA expression, b. TNFRSF10D mRNA expression, c. THBS1 mRNA and protein expression, d. UCHL1 mRNA and protein expression.** The melanoma cell lines were grouped following their methylation profiles: high (>50%), medium (20–50%) and no/low (0–20%).
(PDF)

Figure S4 **Epityper results for the COL1A2, THBS1, TNFRSF10D and UCHL1 promoters in melanocytes, 45 melanoma cell lines, 30 fresh melanoma tumors and cell lines from other tumor types (colon, esophageal and glioma).** The software uses a color coding to show the range of methylation: red to yellow for 0 to 100% of methylation. While the melanocytes show no methylation across the amplicon, the melanoma cell lines and fresh tumors present different patterns of methylation.
(PDF)

Table S1 **Primers for PCR.** Each gene promoter was divided into different amplicons. The target regions were then amplified using the primer pairs and annealing temperatures defined by the MethPrimer program.
(XLS)

Table S2 **Primers for qRT-PCR.**
(XLS)

Table S3 **Levels of gene reactivation in a panel of 12 melanoma cell lines post-5AzadC+TSA treatment.**
(PDF)

Table S4 **Informative CpG sites count.** For each gene, only the amplicons presenting a significant methylation profile difference between melanoma cell lines and melanocytes were scored. In each amplicon, only the informative SpG sites were counted for the final % of methylation value for each gene.
(PDF)
