# Supplementary figures and images for: Cross-Platform Array Screening Identifies COL1A2, THBS1, TNFRSF10D and UCHL1 as Genes Frequently Silenced by Methylation in Melanoma
Source: PLoS One. 2011 Oct 20;6(10):e26121. doi: 10.1371/journal.pone.0026121 (PMC3197591; doi:10.1371/journal.pone.0026121)

Supplementary Figure S1

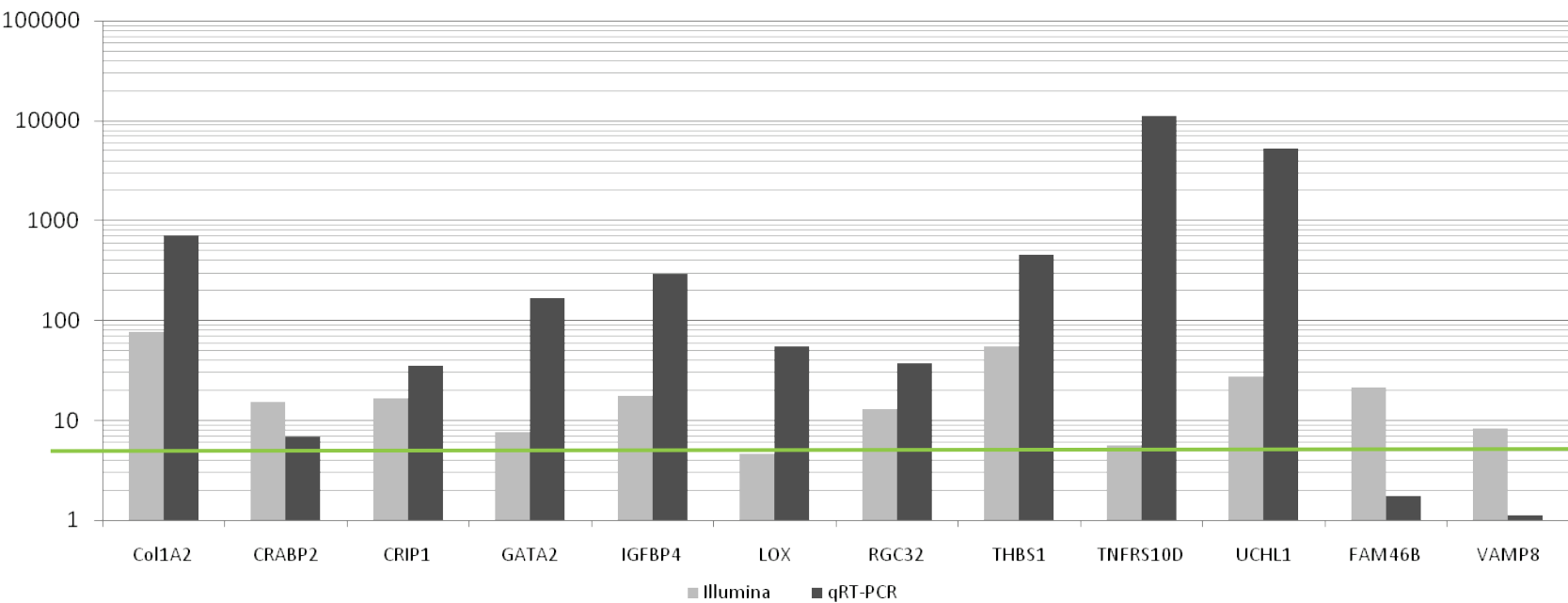

Supplement: Figure S1 — Comparative expression between microarray and qRT-PCR for 12 candidate genes. Plotted are the mean fold-change values for 5 cell lines with the highest expression differences before and after 5Aza-dC treatment. (PDF) [file pone.0026121.s001.pdf]

Supplementary Figure S3

A

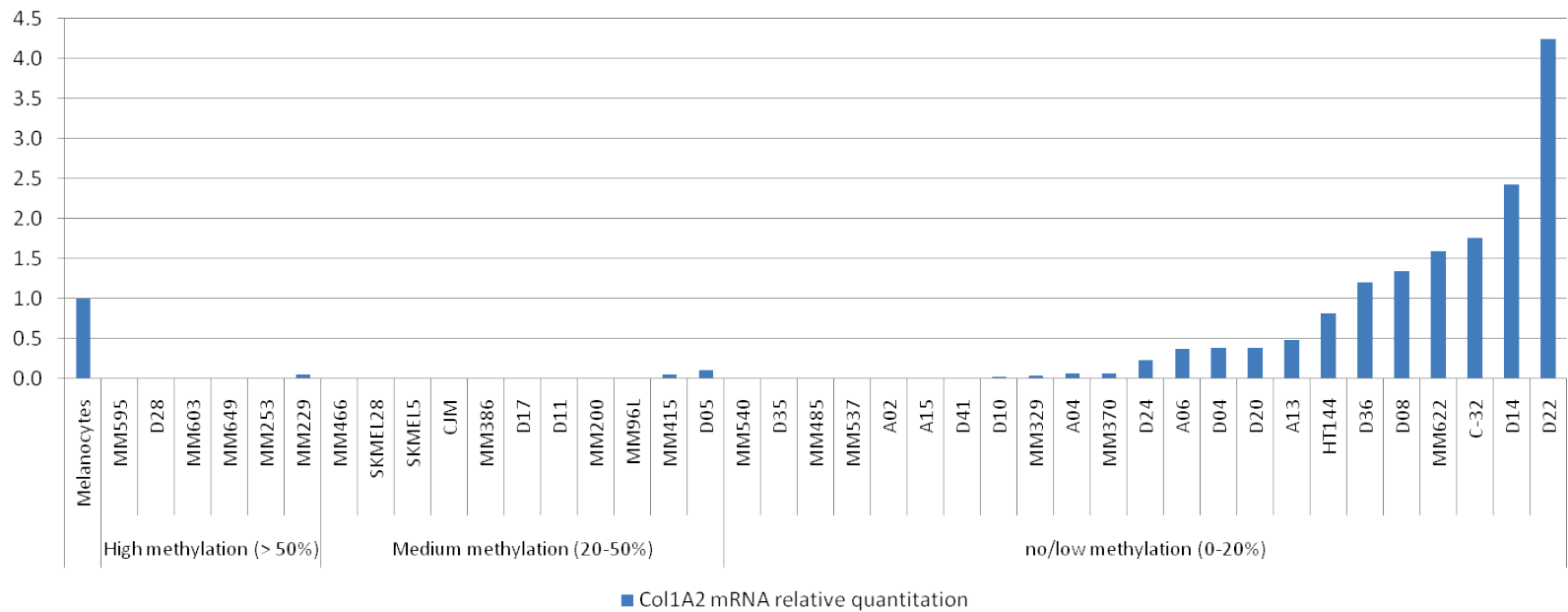

B

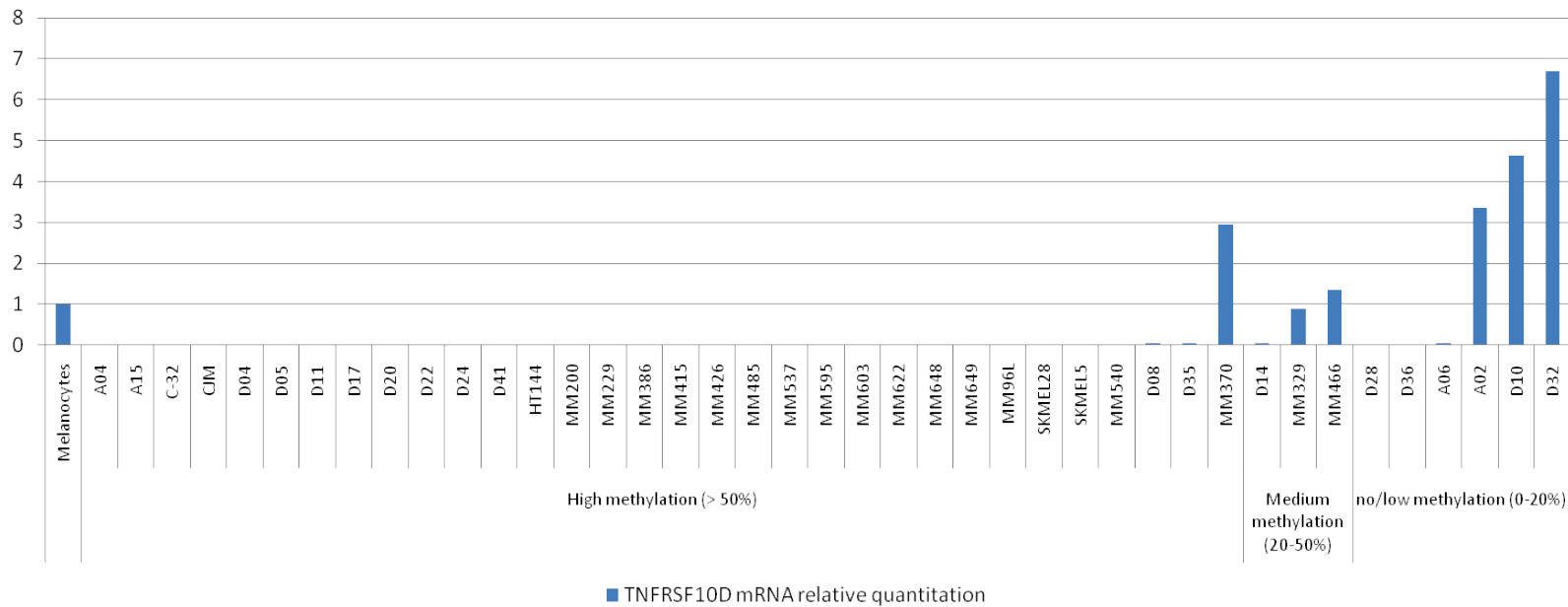

C

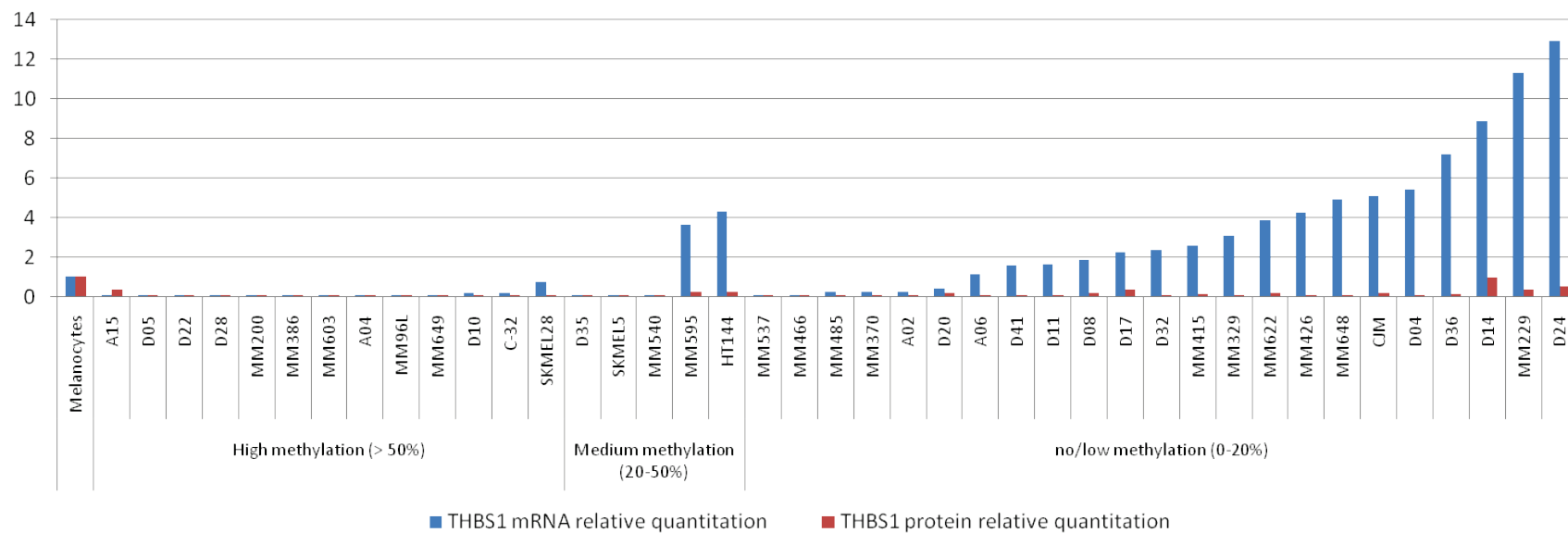

D

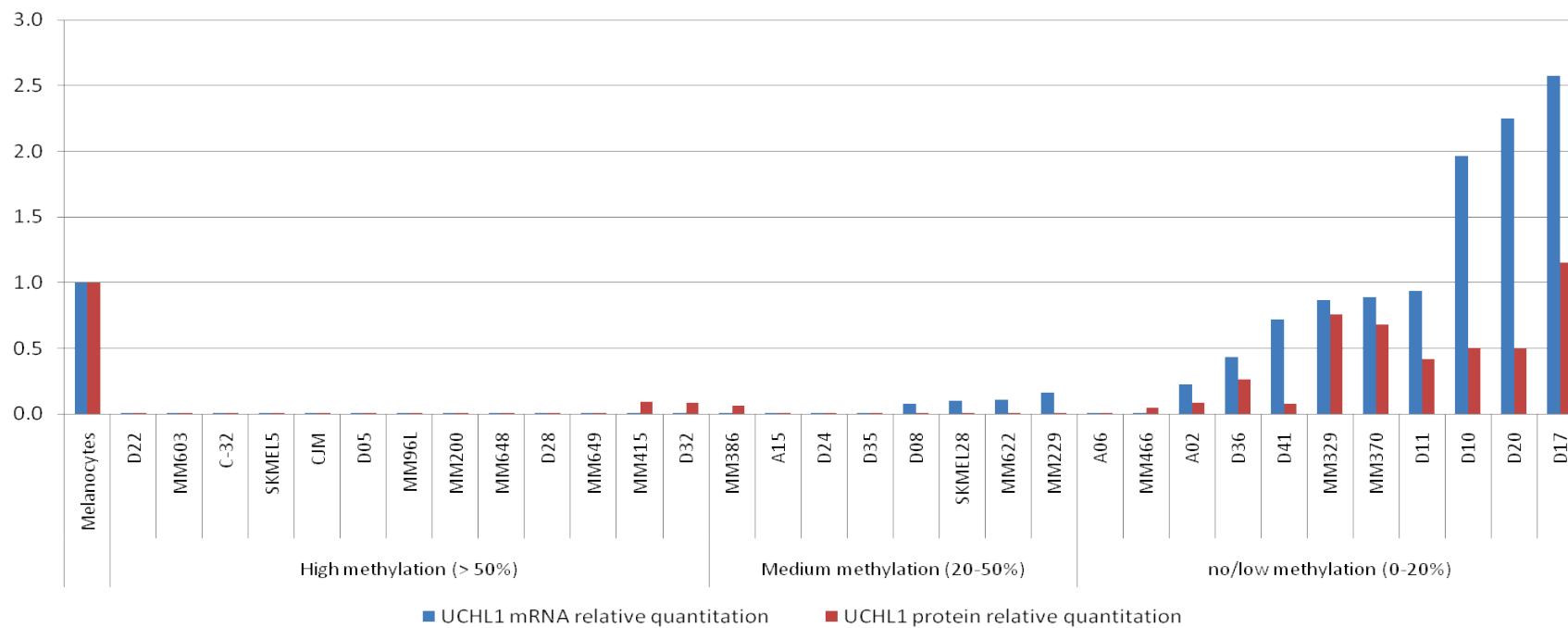

Supplement: Figure S3 — Distribution of the melanoma cell lines according to their methylation status for a. COL1A2 mRNA expression, b. TNFRSF10D mRNA expression, c. THBS1 mRNA and protein expression, d. UCHL1 mRNA and protein expression. The melanoma cell lines were grouped following their methylation profiles: high (>50%), medium (20–50%) and no/low (0–20%). (PDF) [file pone.0026121.s003.pdf]

0% 100% Not analyzed.

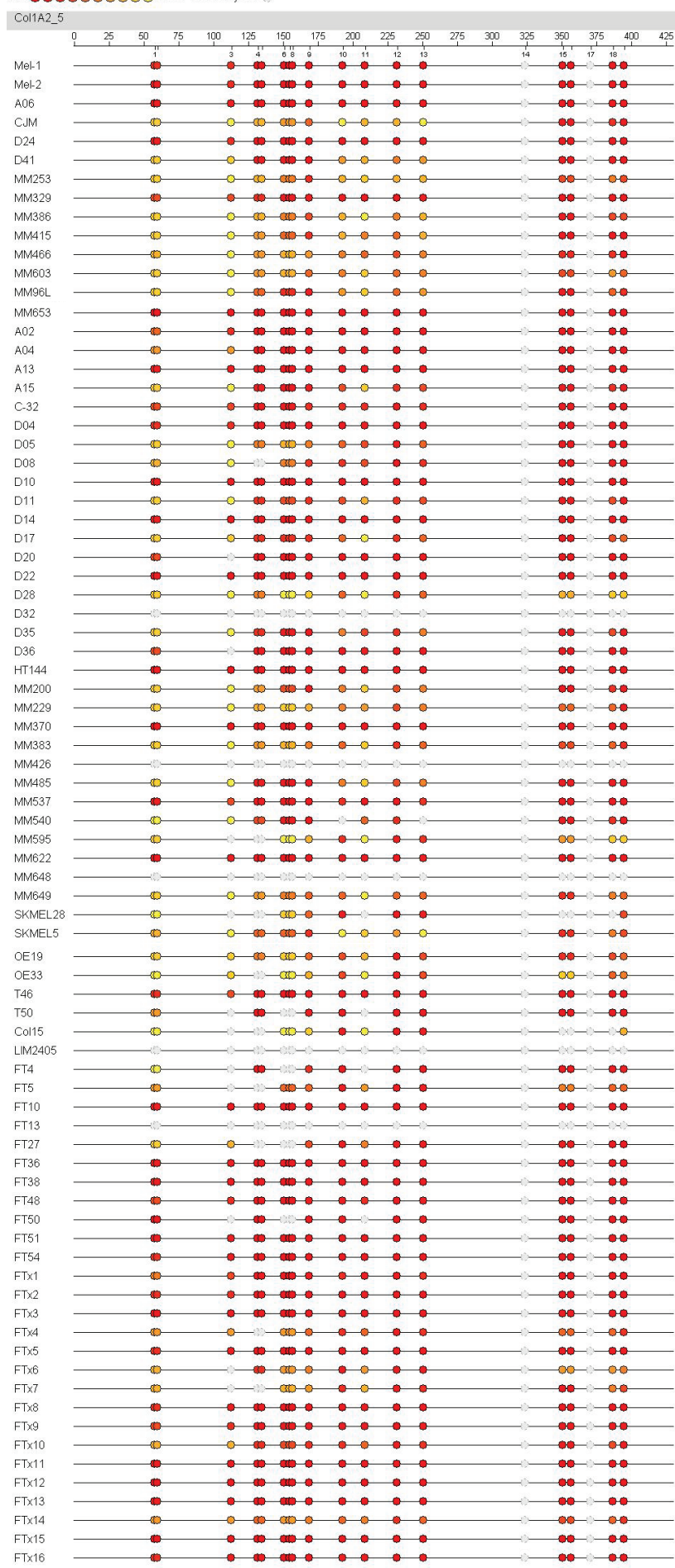

0% 100% Not analyzed

THBS1\_7

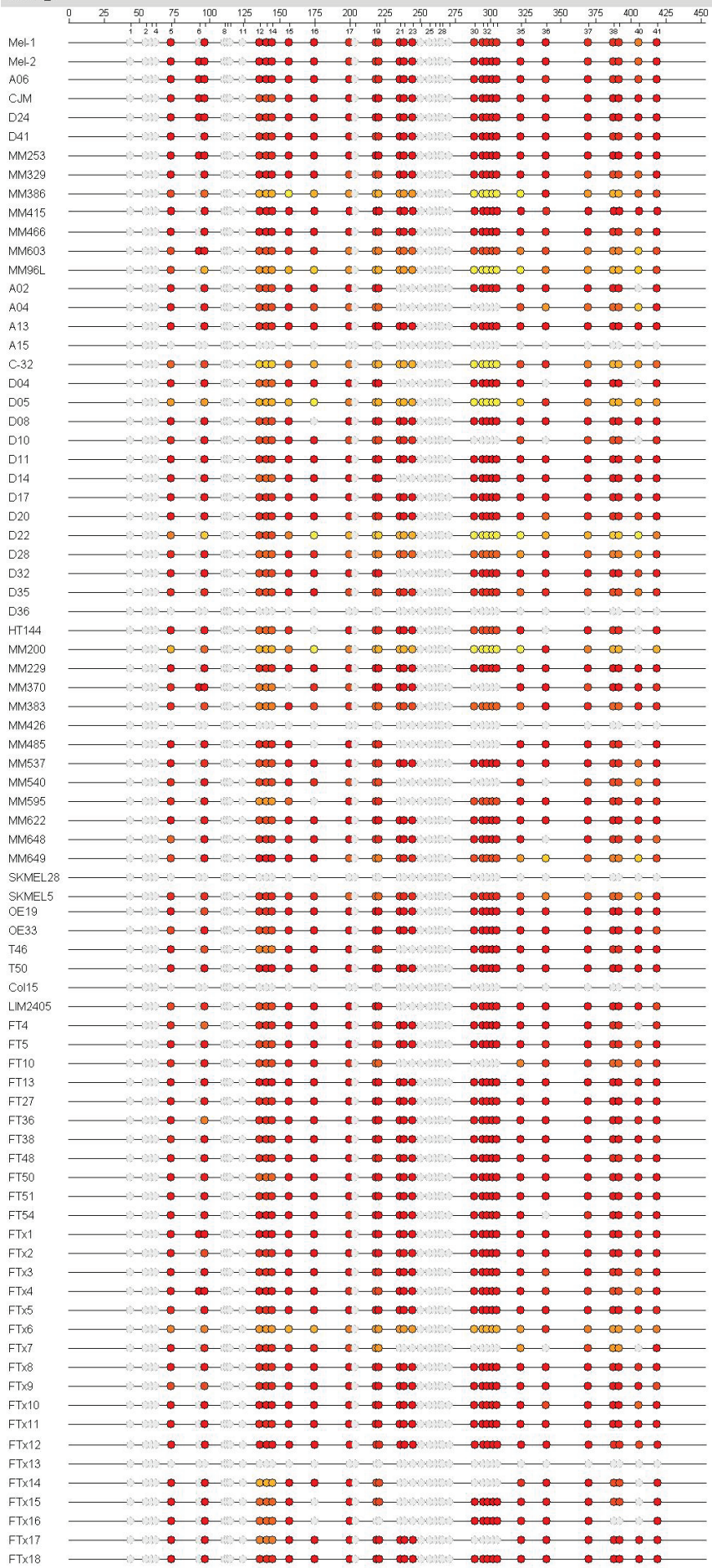

0% 100% Not analyzed

THBS1\_6

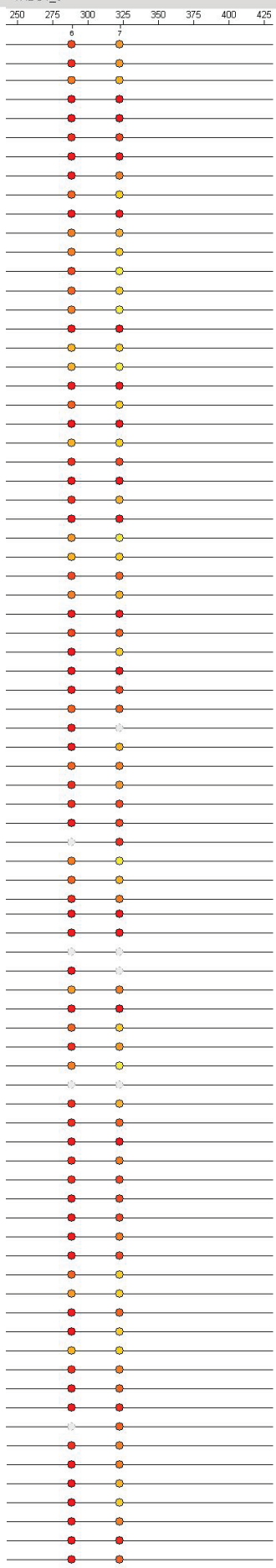

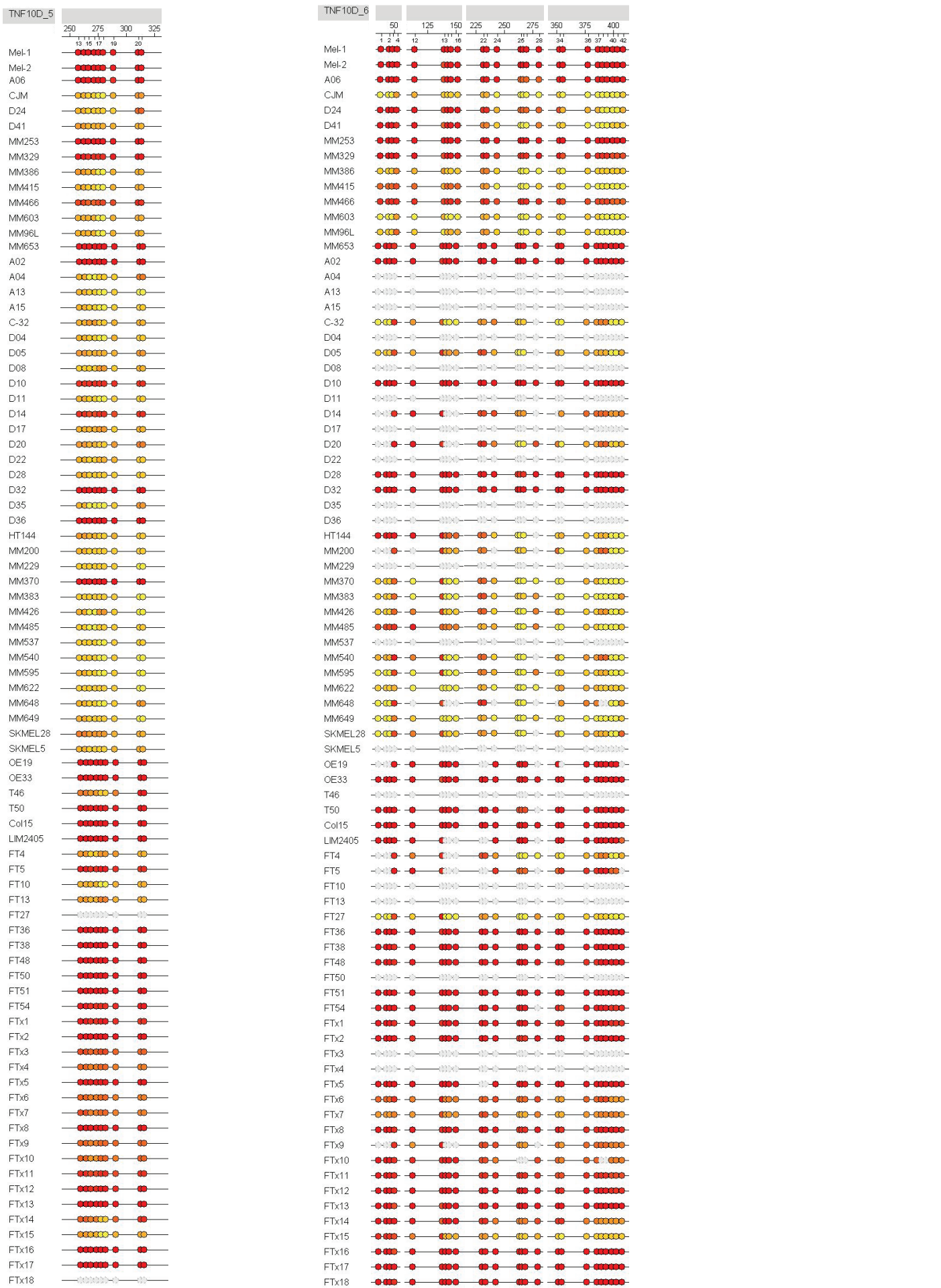

0% 100% Not analyzed

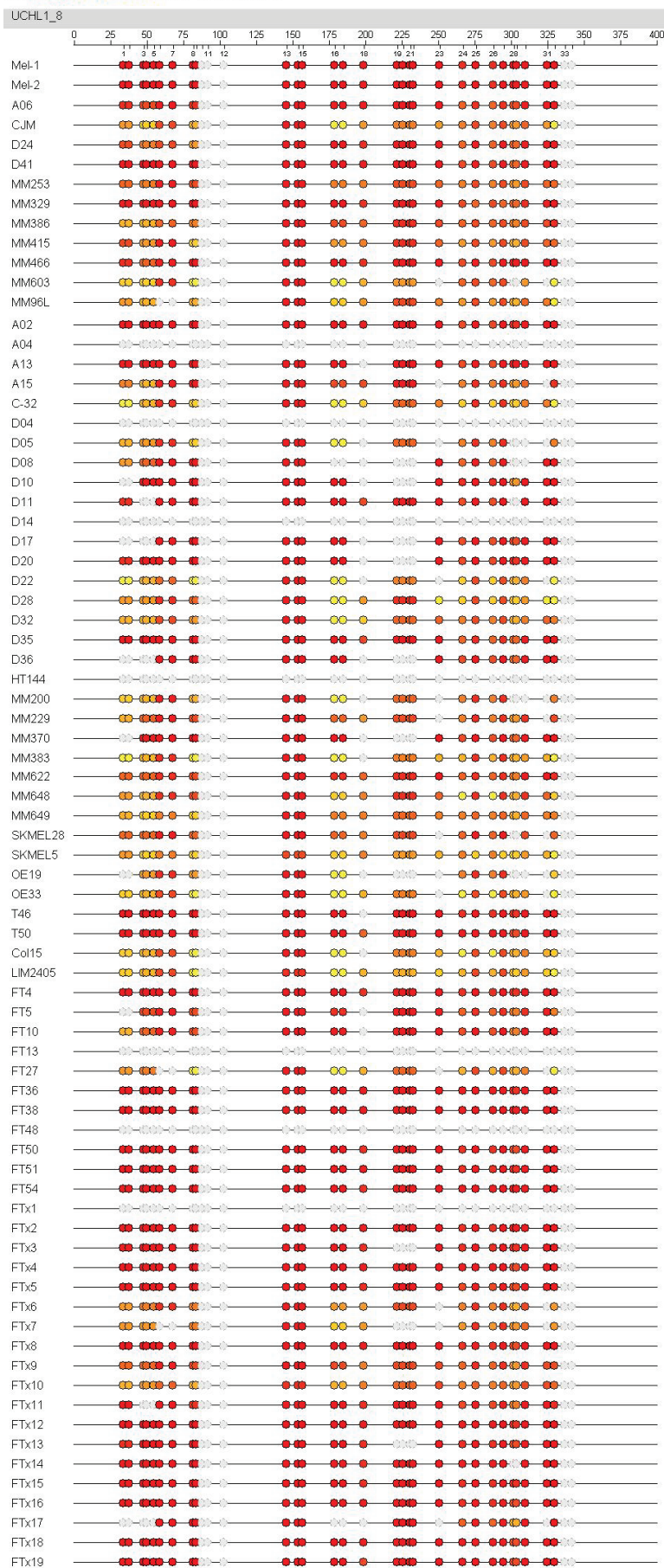

Supplement: Figure S4 — Epityper results for the COL1A2, THBS1, TNFRSF10D and UCHL1 promoters in melanocytes, 45 melanoma cell lines, 30 fresh melanoma tumors and cell lines from other tumor types (colon, esophageal and glioma). The software uses a color coding to show the range of methylation: red to yellow for 0 to 100% of methylation. While the melanocytes show no methylation across the amplicon, the melanoma cell lines and fresh tumors present different patterns of methylation. (PDF) [file pone.0026121.s004.pdf]
