# Supplementary material for: Cross-Platform Array Screening Identifies COL1A2, THBS1, TNFRSF10D and UCHL1 as Genes Frequently Silenced by Methylation in Melanoma
Source: PLoS One. 2011 Oct 20;6(10):e26121. doi: 10.1371/journal.pone.0026121 (PMC3197591; doi:10.1371/journal.pone.0026121)

Col1A2 chr7:94,021,100-94,025,000

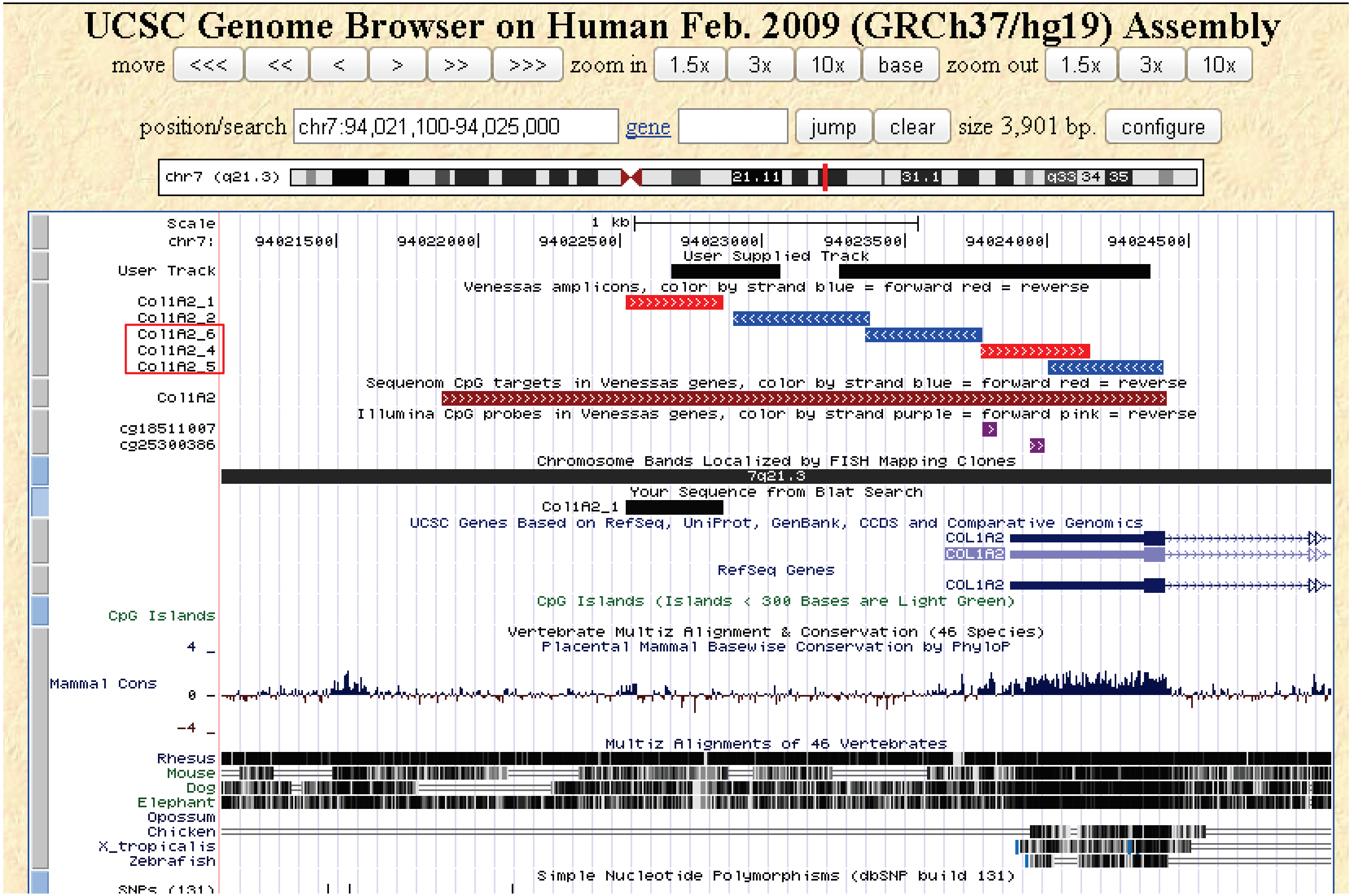

CRIP1 chr14:105,951,800-105,955,600

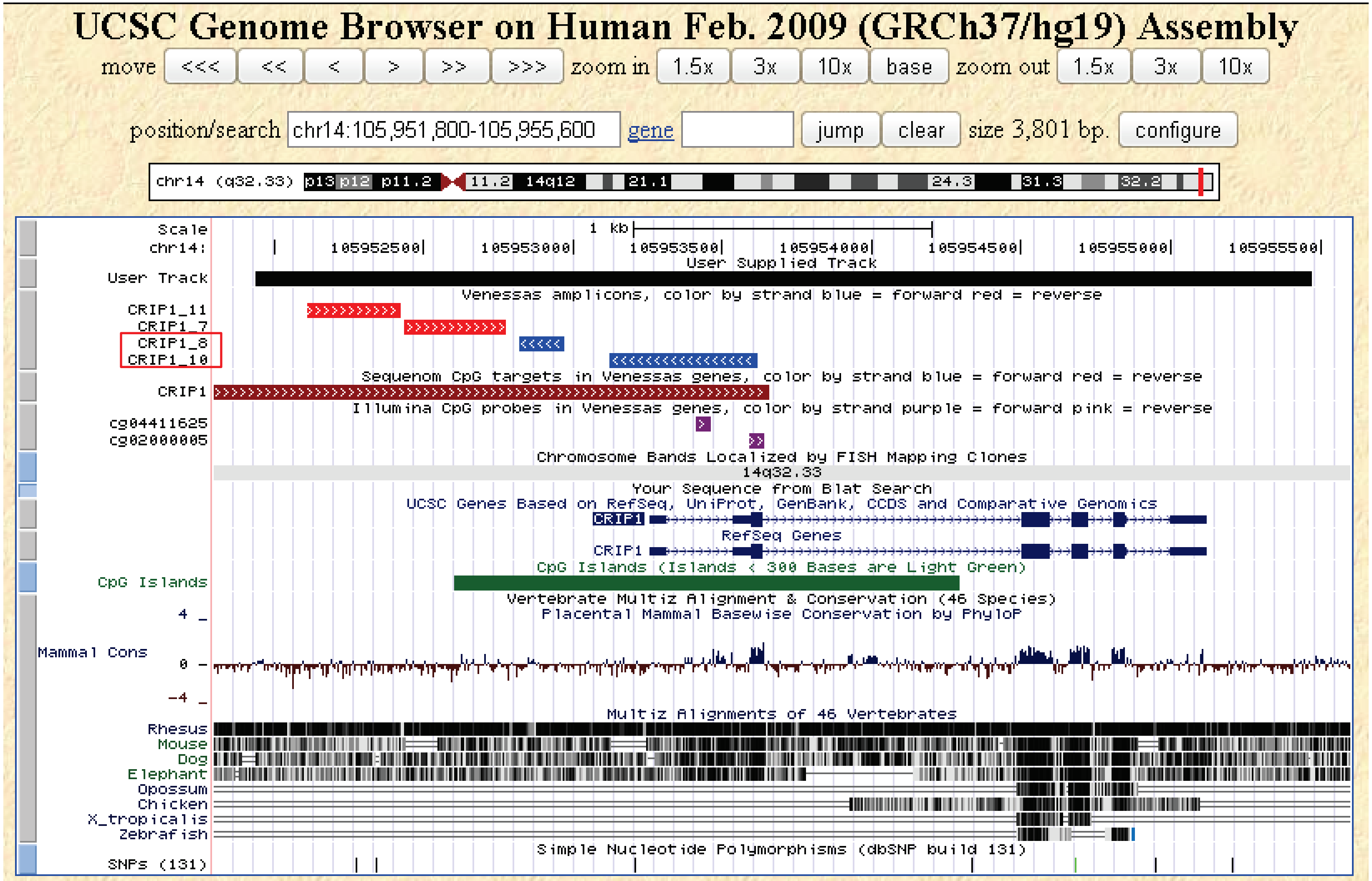

GATA2 chr3:128,204,000-128,217,500

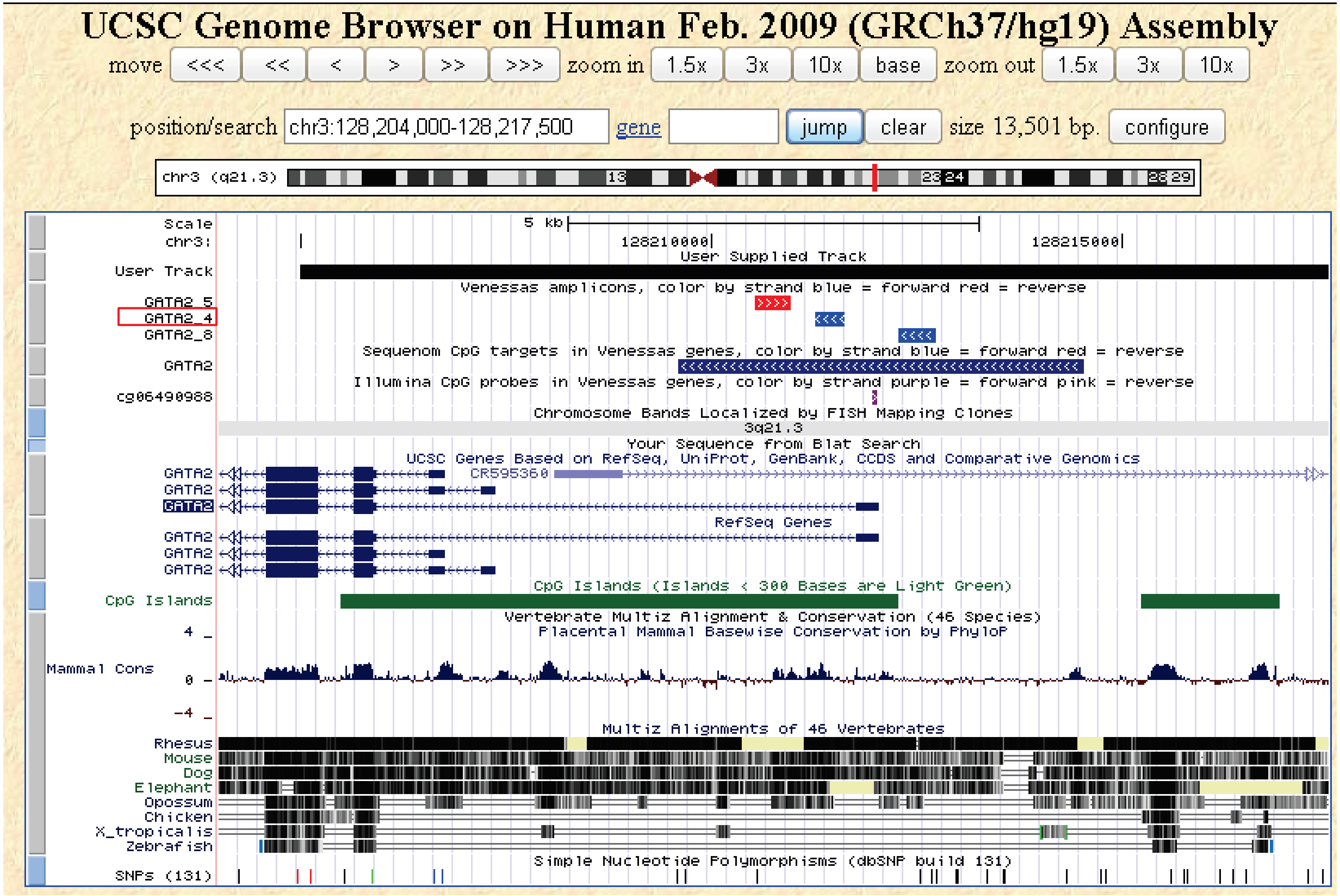

IGFBP4 chr17:38,596,500-38,602,000

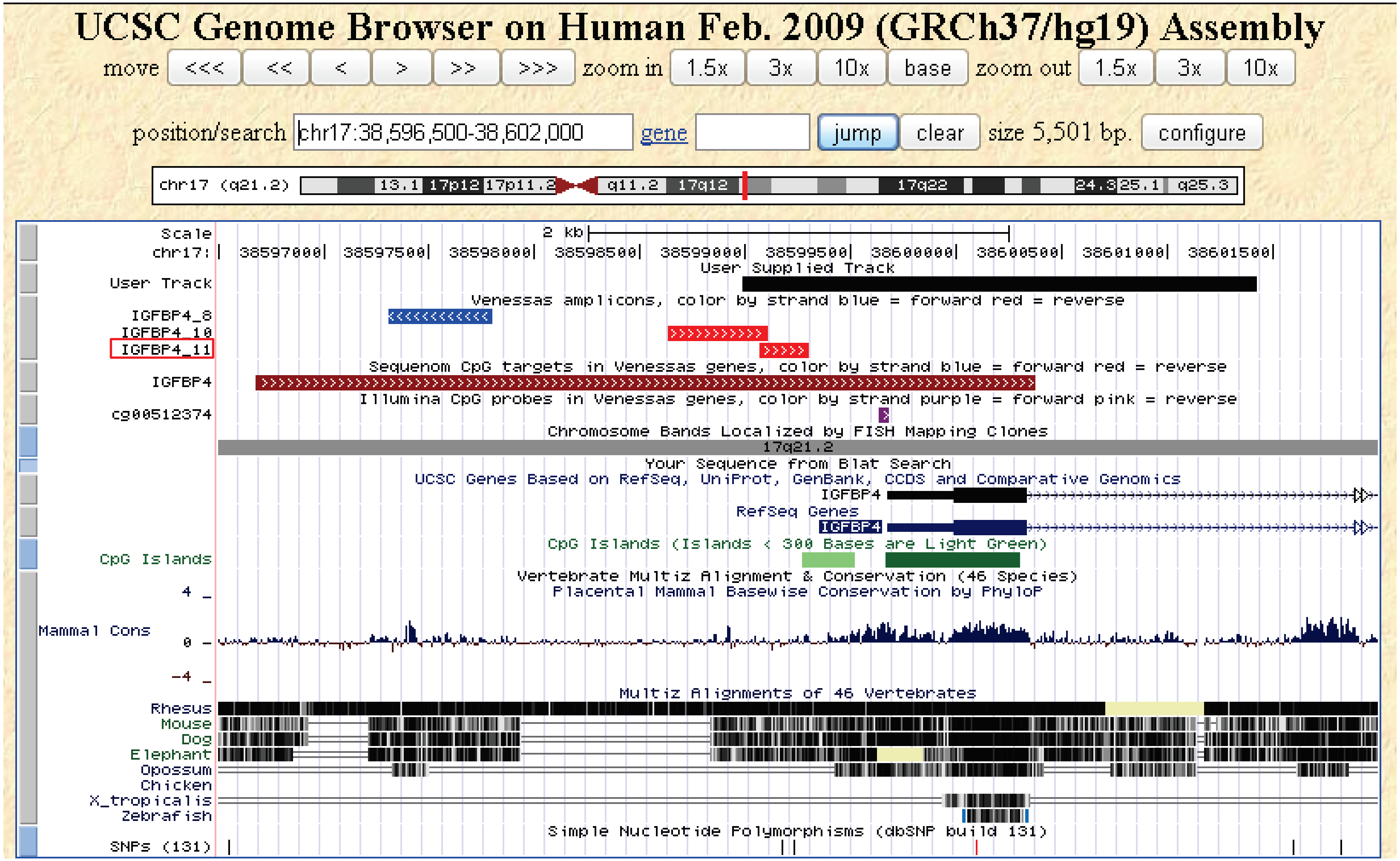

THBS1 chr15:39,870,500-39,876,500

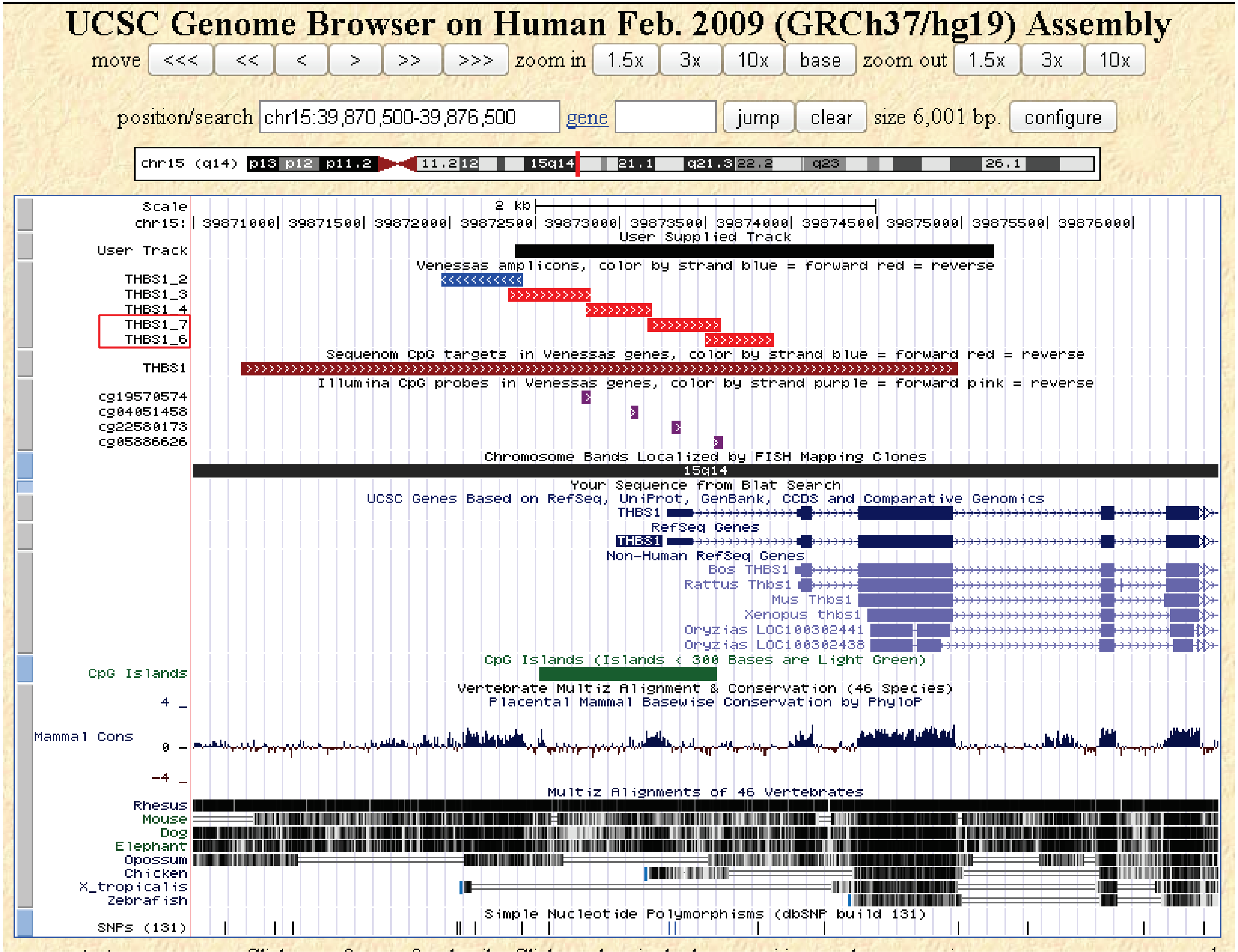

TNFRSF10D chr8:23,020,000-23,025,000

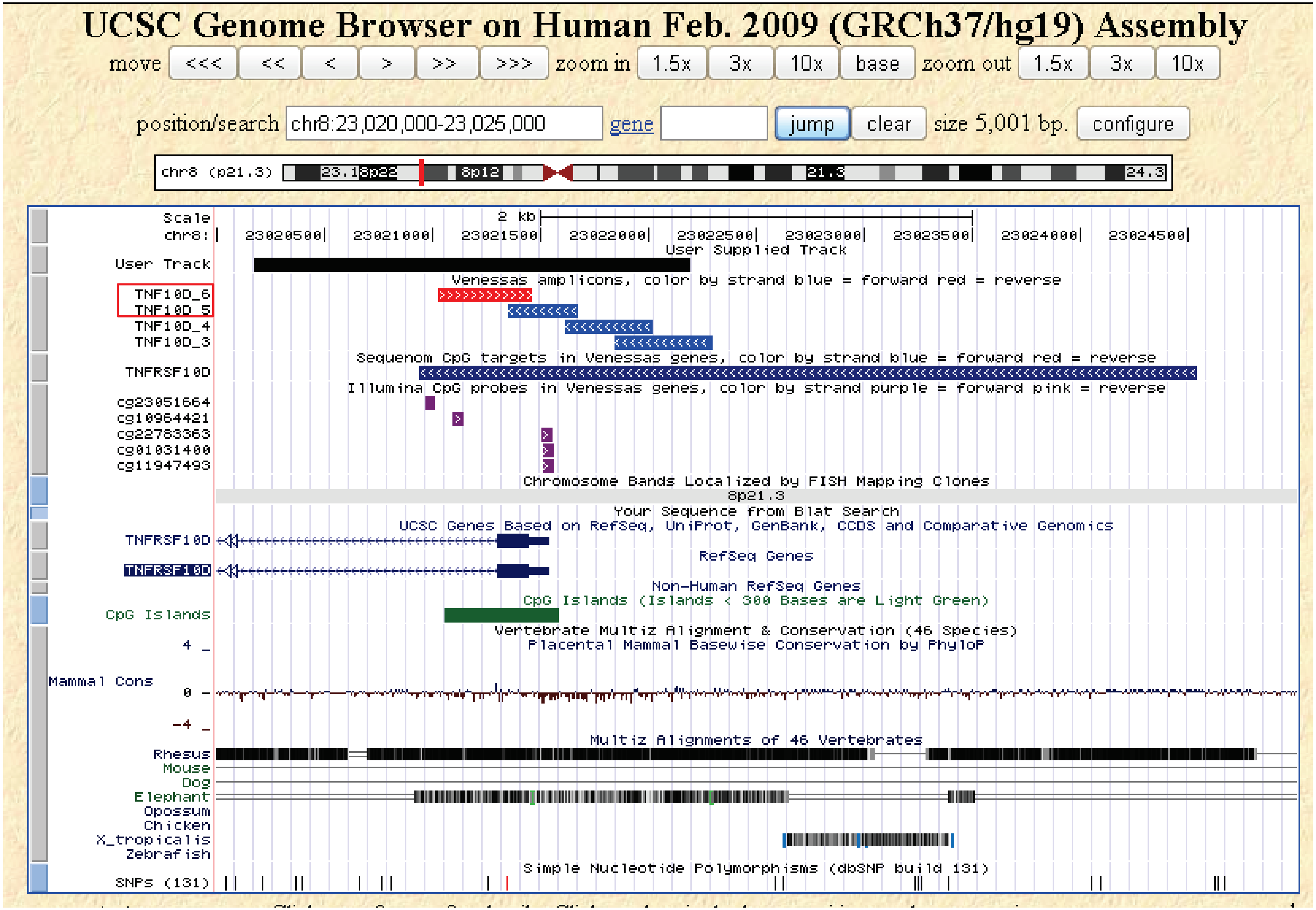

UCHL1 chr4:41,255,500-41,262,000

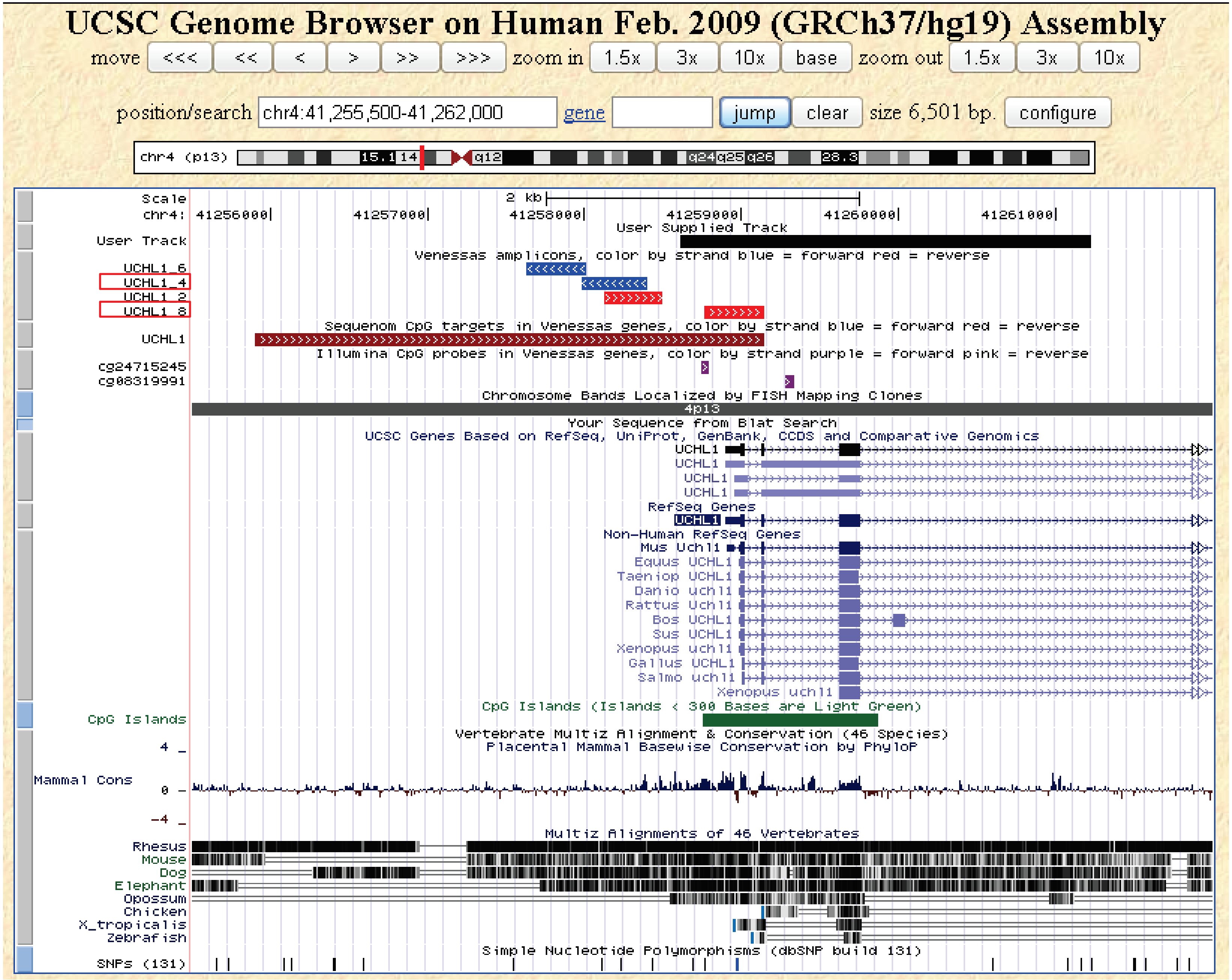

Supplement: Figure S2 — UCSC browser for localisation of the amplicons used for the Epityper assays. The alignments show the localisation of the Illumina probes. (PDF) [file pone.0026121.s002.pdf]
