# Supplementary material for: Cross-Platform Array Screening Identifies COL1A2, THBS1, TNFRSF10D and UCHL1 as Genes Frequently Silenced by Methylation in Melanoma
Source: PLoS One. 2011 Oct 20;6(10):e26121. doi: 10.1371/journal.pone.0026121 (PMC3197591; doi:10.1371/journal.pone.0026121)
Supplement: Table S3 — Levels of gene reactivation in a panel of 12 melanoma cell lines post-5AzadC+TSA treatment. (PDF) [file pone.0026121.s007.pdf]

**Supplementary Table S3**

| Common | Systematic | Genbank | Melanoma cell lines | | | | | | | | | | | | Average fc |
|---|---|---|---|---|---|---|---|---|---|---|---|---|---|---|---|
| | | | AO6 | CJM | D24 | D41 | JAM | MM253 | MM329 | MM386 | MM415 | MM466 | MM603 | MM96L | |
| COL1A2 | ILMN_23060 | NM_000089.3 | | 95.58 | 2.08 | 9.90 | 174.50 | 19.05 | 2.41 | | | 23.86 | 103.70 | 89.06 | 57.79 |
| CRABP2 | ILMN_16252 | NM_001878.2 | 25.99 | 9.29 | 9.48 | 11.09 | 30.98 | 8.97 | 21.07 | 6.63 | 16.30 | 41.68 | 7.45 | 38.61 | 18.96 |
| CRIP1 | ILMN_15885 | NM_001311.3 | 2.24 | 5.46 | | 2.02 | 30.58 | 25.36 | 2.60 | | 14.32 | 4.47 | | 21.78 | 12.09 |
| GATA2 | ILMN_20021 | NM_032638.3 | | 7.17 | 15.79 | 14.97 | 8.75 | 7.45 | 7.33 | 3.23 | 6.56 | 21.12 | 4.24 | 5.31 | 9.26 |
| IGFBP4 | ILMN_9309 | NM_001552.2 | | 16.82 | 3.02 | | 23.84 | 8.39 | 2.90 | 5.16 | 19.79 | 2.60 | 4.48 | 28.34 | 11.53 |
| LOX | ILMN_11693 | NM_002317.3 | | 2.42 | | | 2.81 | 3.38 | | 5.93 | | | | 7.13 | 4.33 |
| RGC32 | ILMN_14549 | NM_014059.1 | 7.34 | 7.65 | 6.23 | 6.29 | 6.67 | 20.32 | 4.35 | 10.83 | 4.53 | 4.66 | 2.54 | 12.90 | 7.86 |
| THBS1 | ILMN_4882 | NM_003246.2 | 3.10 | 3.74 | | | 91.28 | 6.10 | | 82.28 | | 4.12 | 50.21 | 79.76 | 40.07 |
| TNFRSF10D | ILMN_17600 | NM_003840.3 | 2.03 | | 4.63 | | | | | | 12.71 | 5.09 | | 2.97 | 5.49 |
| UCHL1 | ILMN_9422 | NM_004181.3 | | 9.49 | 23.42 | 2.12 | 25.05 | | 2.10 | 6.23 | 13.53 | 11.59 | 24.07 | 70.06 | 18.77 |
