# Supplementary material for: Cross-Platform Array Screening Identifies COL1A2, THBS1, TNFRSF10D and UCHL1 as Genes Frequently Silenced by Methylation in Melanoma
Source: PLoS One. 2011 Oct 20;6(10):e26121. doi: 10.1371/journal.pone.0026121 (PMC3197591; doi:10.1371/journal.pone.0026121)
Supplement: Table S4 — Informative CpG sites count. For each gene, only the amplicons presenting a significant methylation profile difference between melanoma cell lines and melanocytes were scored. In each amplicon, only the informative SpG sites were counted for the final % of methylation value for each gene. (PDF) [file pone.0026121.s008.pdf]

Supplementary Table S4

Genes	# total **amplicons** assessed	# **amplicons** methylated in melanoma cell lines (compared to melanocytes)	Amplicons names	# **CpGs** interrogated in each amplicon	# **CpGs** removed	# **CpGs** informative	# **CpGs** methylated in melanoma cell lines when compared to melanocytes	# **CpGs** counted for the % methylation for each gene
Col1A2	5	3	**Col1A2_4**	18	12	6	2	**19**
			**Col1A2_5**	19	7	12	10	
			**Col1A2_6**	21	9	12	7	
THBS1	5	2	**THBS1_6**	7	2	5	2	**15**
			**THBS1_7**	41	27	14	13	
TNFRSF10D	4	2	**TNF10D_5**	21	7	14	14	**37**
			**TNF10D_6**	42	19	23	23	
UCHL1	4	2	**UCHL1_4**	5	3	2	2	**19**
			**UCHL1_8**	34	14	20	17	
